# A Randomized trial assessing Efficacy and safety of Mineralocorticoid receptor Antagonist therapy compared to Standard antihypertensive Therapy in hypErtension with low Renin (REMASTER): rationale and study design

**DOI:** 10.1038/s41371-024-00931-4

**Published:** 2024-07-18

**Authors:** Sonali S. Shah, Stella May Gwini, Michael Stowasser, Christopher M. Reid, Morag J. Young, Peter J. Fuller, Jun Yang

**Affiliations:** 1 Centre for Endocrinology and Metabolism, Hudson Institute of Medical Research, Clayton, VIC Australia; 2 Department of Endocrinology, Monash Health, Clayton, VIC Australia; 3 Department of Molecular and Translational Science, Monash University, Clayton, VIC Australia; 4 School of Public Health and Preventive Medicine, Monash University, Melbourne, VIC Australia; 5 Endocrine Hypertension Research Centre, University of Queensland Diamantina Institute, Princess Alexandra Hospital, Woolloongabba, QLD Australia

**Keywords:** Clinical trials, Hypertension, Adrenal gland diseases

## Abstract

Low-renin hypertension affects 1 in 4 people with hypertension, but the optimal management of this condition is not known. We hypothesize that a large proportion of people with low-renin hypertension is mediated by excess mineralocorticoid receptor (MR) activation and that targeted treatment with an MR antagonist (MRA) will be beneficial. This randomized, single-blinded, titration-to-effect aims to investigate whether targeted treatment in low-renin hypertension with MRA is better compared to standard antihypertensives in terms of blood pressure control and end-organ protection. Adults with hypertension, who are treatment naïve or are receiving up to two antihypertensive agents and have a low direct renin concentration <10 mU/L will be included. Participants with severe hypertension, a secondary cause of hypertension, pregnant, breastfeeding, with moderate-severe cardiovascular and chronic kidney disease, or on medications that confound interpretation of the plasma direct renin or aldosterone concentrations will be excluded. Eligible participants will be randomized 1:1 to either MRA therapy (spironolactone) or standard anti-hypertensive therapy (perindopril+/− amlodipine) for 48 weeks. Anti-hypertensives will be up-titrated every 12 weeks until target blood pressure is achieved. The primary objective will be to determine the total defined daily dose of antihypertensives required to achieve the target blood pressure and change in mean clinic systolic blood pressure at week 48. Current hypertension guidelines do not have specific recommendations for the choice of anti-hypertensive medications for people with low-renin hypertension. The results of this trial could guide future hypertension guidelines.

## Introduction

Hypertension is a major cause of death worldwide. Despite extensive research into optimal hypertension treatment, blood pressure control remains inadequate with 2 in 3 hypertensive patients not meeting blood pressure targets and often needing multiple anti-hypertensive medications to achieve control [1]. Missing a secondary cause of hypertension and the lack of a stratified approach for the treatment of hypertension have been highlighted as key shortcomings by the 2016 Lancet Commission on Hypertension [2].

Primary aldosteronism is now recognized as the most common cause of secondary hypertension, accounting for 10% of all people with hypertension [3]. Primary aldosteronism is characterized by autonomous aldosterone production in the presence of renin suppression. Under physiological conditions, the renin-angiotensin-aldosterone system plays a pivotal role in the maintenance of blood pressure through sodium and water homeostasis in epithelial tissues. However, in primary aldosteronism, aldosterone production is unregulated resulting in inappropriate activation of the mineralocorticoid receptor (MR) , which results in excess resorption of sodium and water, hypertension, and cardiovascular injury [4]. Diagnosis of primary aldosteronism provides an opportunity for effective treatment with either adrenalectomy or MR antagonists (MRA) such as spironolactone and eplerenone. Targeted treatment in primary aldosteronism with MRA improves blood pressure, and polypharmacy and reduces the elevated risk of cardiovascular disease in this population [5].

Importantly, primary aldosteronism is part of a renin-independent hyperaldosteronism continuum. Recent studies have reported the correlation between low renin, markers of increased MR activity, and progressive development of incident hypertension [6]. As such, people with a low direct renin concentration and various levels of aldosterone concentration that do not result in an aldosterone-to-renin ratio that meets the ‘positive’ screening threshold for primary aldosteronism are part of the continuum. Traditionally referred to as low-renin hypertension or low-renin essential hypertension, this condition may be present in up to 30% of people currently labelled as having essential hypertension [7, 8]. A recent meta-analysis comparing MRA to other antihypertensives has shown that MRA is more effective in lowering systolic blood pressure compared to commonly used first-line standard antihypertensives [9–11]. In addition, observational data suggests that MRA use in low-renin hypertension reduces end-organ dysfunction, for example, left ventricular hypertrophy and albuminuria [12]. Despite evidence supporting treatment that differs from standard practice, low-renin hypertension as a condition distinct from essential hypertension is overlooked due to renin and aldosterone concentrations being neither routinely requested nor used to guide initial therapy [10, 11]. The retrospective diagnosis of low-renin hypertension is challenging as many commonly used antihypertensives interfere with direct renin concentration interpretation [5]. At present, an add-on antihypertensive treatment strategy is recommended by guidelines, and MRA treatment is reserved as a fourth-line agent for resistant hypertension [10, 11]. We may therefore be missing an important opportunity to intervene early to provide targeted treatment with spironolactone, a widely used MRA that is readily available and inexpensive.

Personalizing treatment to the underlying pathophysiology for an individual is likely to improve blood pressure control with fewer medications and reduce/prevent end-organ damage [13]. However, translation to clinical practice has been limited due to concerns regarding the tolerability of MRA therapy and older clinical trials showing conflicting results likely due to suboptimal research methodology. Our study aims to address some of these limitations by conducting a single-blinded randomized controlled trial comparing targeted treatment with MRA versus commonly used standard antihypertensives in adults with low-renin hypertension [14]. We hypothesize that in a large proportion of patients with low-renin hypertension, the underlying pathophysiology involves MR activation and therefore targeted treatment with an MRA will offer greater antihypertensive effects and end-organ protection. This Randomized trial assessing the Efficacy and safety of Mineralocorticoid receptor Antagonist therapy compared to Standard antihypertensive Therapy in hypErtension with low Renin (REMASTER) therefore seeks to evaluate the efficacy and safety of MRA therapy in participants with low-renin hypertension and to evaluate the clinical, biochemical, and molecular markers that determine MRA responsiveness in people with low-renin hypertension.

## Methods and analysis

Reporting of the methods follows the SPIRIT guidelines [15].

### Trial design and study setting

The trial will be a randomized, single-blinded, active-controlled, titration-to-effect, pragmatic study comparing MRA to standard anti-hypertensive treatment for participants with low-renin hypertension referred to the Endocrine Hypertension Clinic at Monash Health, Victoria’s largest public health service. The clinic receives referrals from both general practitioners and specialists. Before performing any trial-specific procedures, a signed consent form will be obtained from each participant. The investigator will confirm that the participant comprehends the information provided and answer any questions about the trial. When the inclusion/exclusion criteria have been addressed and the eligibility of the participant confirmed, the participant will then be randomized 1:1 to one of the trial arms. All participants will undergo a seated saline suppression test to exclude primary aldosteronism. Renin concentration exists on a continuum. The definition of low-renin hypertension is somewhat arbitrary and has traditionally been defined as a plasma renin activity <1 ng/ml/h which approximately equates to a direct renin concentration of 8–12 mU/L. Furthermore, in an eight-week monotherapy study by Weinberger, participants with direct renin concentration <9.4 mU/L had a better blood pressure response to eplerenone compared to losartan: mean change in systolic blood pressure of –17.2 mmHg versus –5.4 mmHg [16]. We therefore selected a threshold of <10 mU/L to define low-renin hypertension.

### Trial population

#### Inclusion criteria

Adult ambulatory participants with hypertension, defined as a mean seated blood pressure >140/90 mmHg, and are treatment-naive or with any blood pressure level on up to two antihypertensive agents, have a low direct renin concentration <10 mU/L (measured on non-interfering medications), but who do not have primary aldosteronism or monogenic forms of low-renin hypertension, and can provide informed consent to participate in the trial.

#### Exclusion criteria

See Table 1.

**Table 1:** Eligibility criteria.

Inclusion criteria	-Hypertension, defined as a mean seated blood pressure >140/90 mmHg and is treatment naïve or any blood pressure if receiving up to two antihypertensive agents. -Low direct renin concentration <10 mU/L (measured on non-interfering medications) -Can provide informed consent.
Exclusion criteria	-Blood pressure >180/120 mmHg. -On mineralocorticoid receptor antagonist treatment or medications confounding renin or aldosterone measurement e.g., glucocorticoids, oral estrogen, and sodium-glucose co-transporter 2 inhibitors. -Pregnant, breastfeeding or women of childbearing potential not on reliable contraception. -Hyperkalemic, potassium >5.0 mmol/L. -History of uncontrolled diabetes with glycosylated hemoglobin >7.5%, chronic kidney disease with an estimated glomerular filtration rate <50 mL/min/1.73 m^2^, heart failure class II–IV, ischemic heart disease, transient ischemic attack, stroke, or atrial fibrillation. -History of liquorice abuse or other secondary causes of hypertension such as primary hyperparathyroidism, primary aldosteronism, autonomous cortisol secretion, hyperthyroidism, phaeochromocytoma, renal artery stenosis or known monogenic causes of low-renin hypertension. -Known hypersensitivity to the trial medications.

### Trial procedure

**Fig. 1:**
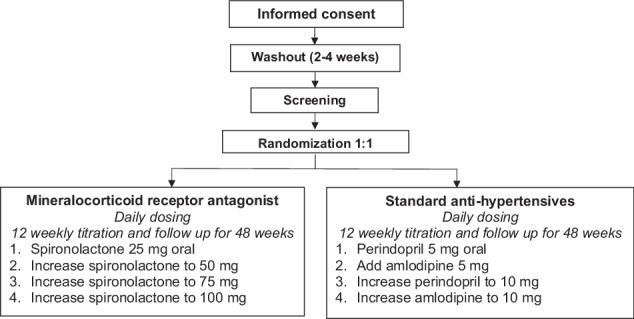
Titration steps in the treatment algorithm.

Study participants meeting the inclusion and exclusion criteria will be randomized 1:1 via REDCap to either MRA, spironolactone (intervention), or standard care, perindopril+/-amlodipine (control), using a block randomization method. The schedule has been prepared by a biostatistician and uploaded to REDCap and is not available to the clinic staff. Participants on antihypertensive treatment at baseline will undergo a washout period of 2–4 weeks before screening and randomization. If there are clinical concerns, prazosin, a short-acting antihypertensive can be continued until 48 h pre-randomization. The standard care group will receive treatment as per local prescribing patterns and hypertension guidelines [14]. Antihypertensive medications will be titrated as required at 12-weekly intervals until target blood pressure of <140/90 mmHg is achieved in both groups and renin is unsuppressed i.e., >15 mU/L in the MRA group (Fig. 1). The biochemical titration target for the MRA group is based on evidence that treatment adequacy is associated with normalized renin [17]. Participants will be reviewed every 6–12 weeks and treated for a total of 48 weeks. Administration of medication will be standardized to night-time dosing once daily for consistency. Blood pressure measurements will be taken seated in triplicates using an automated blood pressure machine (Omron) and the average of 2nd and 3rd readings will be calculated. Automated office blood pressure measurement has been shown to correlate with out-of-clinic measures [18].

Pathology will be collected at Monash Health Pathology laboratories. Both aldosterone and direct renin concentration will be measured using chemiluminescent immunoassays (LIAISON, DiaSorin). For the seated saline suppression test, aldosterone concentration will be measured using mass spectrometry as well due to its greater accuracy [19]. Primary aldosteronism will be excluded if aldosterone concentration post saline infusion is <162 pmol/L [20].

### Compliance

Medication compliance will be assessed at each study visit using medication diaries and pill counting when medications are returned to the pharmacy.

### Relevant concomitant therapies allowed and excluded

Women of childbearing age will need to be on effective contraception given the potential for teratogenicity of the trial medications during study participation and until 2 months after the last dose of trial medications. Trial participants needing to use medications that can interfere with the measurement of blood pressure, renin, or aldosterone during the treatment and follow-up phase of the trial will be excluded.

### Safety considerations

All medications used in the trial have been entered into the Australian Register of Therapeutic Goods. Spironolactone, perindopril, and amlodipine are approved by Therapeutic Guides Australia for the treatment of hypertension. The following adverse effects will be noted at each visit: gynecomastia, breast pain, reduced libido, menstrual irregularity, cough, hyperkalemia, and an increase of >30% in serum creatinine.

Adverse effects will be described in the patient’s medical record as follows: a description of symptoms including duration, severity (mild, moderate, or severe), seriousness (i.e., is it a serious adverse event?), any action taken, the outcome and likelihood of the relationship to the trial treatment (unrelated, possible, probable, definite) [21]. All serious adverse effects that occur from the time the participant signs the consent form, up to and including the last scheduled visit, will be reported within 24 h to an independent Data and Safety Monitoring Board, consisting of four members including a biostatistician and doctors with expertise in clinical trials. A trial progress update will be sent quarterly to the Data and Safety Monitoring Board. Trial conduct, protocol compliance, and safety are under their oversight.

### Dose and medication modification

If a participant on spironolactone treatment develops troublesome gynecomastia, breast tenderness, or irregular menses, they will be prescribed eplerenone in place of spironolactone at the same dose but twice a day e.g., participants on spironolactone 25 mg daily will be switched to eplerenone 25 mg twice a day. Eplerenone is a less potent but more selective MRA with minimal effect on the progesterone and androgen receptors. If perindopril 5/10 mg is not tolerated due to side effects such as cough, participants will be prescribed irbesartan 150/300 mg, respectively. Participants requiring a change in medication to eplerenone or irbesartan will be unblinded but will remain in the study and contribute to the final analysis. If a participant develops hyperkalemia, defined as serum potassium > 5.5 mmol/L on a non-hemolyzed blood sample, or has a 30% increase in creatinine due to perindopril/irbesartan or spironolactone/eplerenone, the dose of the trial mediation will be reduced to the highest tolerated dose.

### Blinding

Participants will be blinded to the treatment by using encapsulating medications through a third party. The nurse and technician performing clinic blood pressure, and transthoracic echocardiograms will also be blinded to reduce any potential bias in the reporting of outcomes. Clinicians prescribing the antihypertensives will not be blinded to treatment allocation as the titration protocol is treatment-specific. Although a possible limitation, we anticipate that any treatment bias will be minimal given that the prescribed treatment will be driven by the study protocol and up-titration of treatments will be based on clinic blood pressure measurements performed by the nurse (blinded to treatment allocation), and direct renin concentration values measured at the hospital laboratory (for the MRA arm only).

### Study objectives

The primary objective will be to assess the change in mean clinic systolic blood pressure from baseline to week 48 and to determine the total defined daily dose of antihypertensives required to achieve the target blood pressure. The ‘defined daily dose’ is described by the World Health Organization as the average maintenance dose per day for a drug used for its main indication in adults and is a measure of drug utilization [22]. The defined daily dose of spironolactone is 75 mg/day, perindopril 5 mg/day, and amlodipine 5 mg/day [22].

The secondary and exploratory objectives of this study are summarized in Table 2.

**Table 2:** Study objectives.

Primary	To evaluate the -change in mean seated clinic systolic blood pressure from baseline to week 48 compared with standard antihypertensive treatment. -total defined daily dose of antihypertensives required to achieve target blood pressure with MRA compared to standard antihypertensive treatment.
Secondary	To evaluate the effect of MRA compared with standard antihypertensive treatment on the: -change in mean seated clinic diastolic blood pressure from baseline to week 48 compared with standard antihypertensive treatment. -change in mean seated clinic systolic and diastolic blood pressure from baseline to week 24. -change in systolic and diastolic blood pressure on ambulatory blood pressure monitoring from baseline to week 48. -time to achieve the target blood pressure. -the proportion of participants that achieved target blood pressure at week 48. -change in the left ventricular mass index and global longitudinal strain on transthoracic echocardiogram from baseline to week 48. -change in endothelial function measured by EndoPAT (Itamar Medical, Israel) from baseline to week 48. -change in 24-h urinary microalbumin excretion from baseline to week 48. -the proportion of participants that discontinued trial medication due to adverse effects. -the proportion of participants who developed hyperkalemia. -change in the quality of life assessed using the SF-36 Quality of Life Questionnaire from baseline to week 48.
Exploratory	To evaluate characteristics that predict response to MRA including age, ethnicity, sex, baseline PAC, DRC, ARR, urinary aldosterone, urinary sodium, post-saline suppression test PAC (measured by LCMS and radioimmunoassay), monocyte-based transcriptomic markers and plasma and urinary steroid profiles.

*ARR* aldosterone to renin ratio, *LCMS* liquid chromatography-tandem mass spectrometry, *MRA* mineralocorticoid receptor antagonist, *PAC* plasma aldosterone concentration, *DRC* direct renin concentration.

### Data collection and management

The medical history, examination findings, medication side effects, compliance, and investigation results will be collected at each study visit (Table 3) and entered via the secure web-based data management system, REDCap. Data entry will be performed by the investigators. Data logic and consistency checks are programmed into the data entry forms so that data entry errors can be caught in real time, and queries are auto-generated. Data generated will be held in strict confidence. No information concerning the trial or the data will be released to any unauthorized third party without the prior written approval of the Hudson Institute of Medical Research.

**Table 3:** Data collection schedule.

		Week						
	–6	–2	0	6	12	24	36	48
**Enrolment**								
*Informed consent*	x							
*DRCx2, PACx2, ARR x2, UEC, BhCG (if relevant), HBA1C, PTH, calcium, vitamin D, TSH, plasma metanephrines, 24-h urinary cortisol, and saline suppression test*.		x						
*Randomization*			x					
**Intervention**								
*Active: MRA*								
*Control: standard antihypertensives*								
**Assessment**								
*BMI, age, sex, ethnicity, smoking status, ethanol intake, OSA screening*			x					x (weight only)
*Mean clinic blood pressure*	x		x	x	x	x	x	x
*PAC, DRC, ARR, UEC*				x	x	x	x	x
*Fasting glucose, fasting lipid profile, liver function tests*		x						x
*PTH, calcium, vitamin D*								x
*24-h urine sodium, potassium, calcium, aldosterone, and albumin*		x						x
*24-h urine steroid profile*		x						
*FBE, CRP, BNP*		x						x
*Plasma steroid profile*		x						
*Plasma and urine biobank sample*		x						x
*Monocyte gene expression profile*		x						
*ABPM*			x					x
*TTE*			x					x
*Endothelial function*			x					x
*QOL (SF-36 questionnaire)*			x					x
*Compliance assessment*				x	x	x	x	x
*Adverse effect monitoring*				x	x	x	x	x

*ABPM* ambulatory blood pressure monitoring, *ARR* aldosterone to renin ratio, *BhCG* beta-human chorionic gonadotrophin, *BMI* body mass index, *BNP* brain natriuretic peptide, *CRP* C-reactive protein, *FBE* full blood examination, *GLS* global longitudinal strain, *HBA1c* glycosylated hemoglobin, *LFT* liver function tests, *LVMI* left ventricular mass index, *MRA* mineralocorticoid receptor antagonists, *OSA* obstructive sleep apnea, *PAC* plasma aldosterone concentration, *DRC* direct renin concentration, *PTH* parathyroid stimulating hormone, *SST* saline suppression test, *TSH* thyroid-stimulating hormone, *TTE* trans-thoracic echocardiogram.

### Biological specimen collection and storage

Blood and urine biospecimens collected at baseline and the end of the trial will be frozen and stored at Monash Pathology and may be retrieved to measure new markers of low-renin hypertension and any subtypes identified in this trial. All other biospecimens will be discarded.

### Sample size calculation and statistical analysis

The sample size estimation will be based on the two primary objectives. In a study of hypertensive patients, Weinberger et al showed that after 16 weeks of treatment, systolic blood pressure among patients with low-renin hypertension (renin <9.4 mU/L) was reduced by 17.2 mmHg in the eplerenone group compared with 5.4 mmHg in the losartan group (no standard deviations were reported) [16]. Assuming a similar drop in systolic blood pressure between treatment groups with an equal standard deviation of 10 mmHg for the change in blood pressure, a minimum sample size of 24 patients per arm would be required. For the second primary endpoint of the defined daily dose required to achieve target blood pressure, a comprehensive systematic review of the literature was conducted, but no previous trials reported the expected defined daily dose for either spironolactone or perindopril in low-renin hypertension. Therefore, in the absence of such information, a large effect size will be assumed (Cohen’s d = 0.8). For this effect size, a minimum sample size of 50 patients per group is needed. The two sample size estimations account for 90% power in the study, with a Bonferroni family-wise error rate of 0.025 (two-sided) and a 20% attrition. Bonferroni multiplicity was considered because the outcomes from this study would be confirmative, and the defined daily dose and blood pressure change are presumed to be closely related outcome measures for blood pressure control. Hence the target sample size for this study is 100 patients (50 randomized to each arm of the study).

Participants will be recruited from the Endocrine Hypertension Clinic at Monash Health. In addition to referrals from other specialists, the Endocrine Hypertension research group has established a relationship with over 40 general practice clinics and 200 practitioners who routinely refer patients to the clinic.

Analysis of the efficacy of each intervention will be performed on an intention-to-treat basis. All randomized patients with a baseline assessment and at least one post-baseline assessment will be included in the statistical analysis. Safety analyses will include all randomized patients who received at least one dose of study medication. An additional per-protocol analysis will be considered in the case of non-compliance and/or medication non-adherence. All data will be analyzed as observed and no data imputation is anticipated unless significant missing data is observed.

A generalized linear model will be used to conduct a between-group comparison for the systolic blood pressure, defined daily dose of antihypertensives, left ventricular mass index, global longitudinal strain, endothelial dysfunction, and 24-h urinary microalbumin excretion. If some participants do not achieve target blood pressure, the data will be treated and analyzed using methods for right censored data and between groups comparison will be done using Kaplan–Meier curves and the log-rank test. The difference in the change in blood pressure between groups will be estimated based on the interaction between the time and group variables using a mixed-effects model. A Cox proportional hazards model will be used to assess and compare the time to achieve target blood pressure in the MRA and standard treatment groups. The chi-squared test will be used for between-group comparison of the proportion of participants who discontinued treatment due to adverse effects or developed hyperkalemia during the treatment period. Data will be analyzed using Stata Statistical Package version 17 or later, R Statistical Package version 3 or later, or SPSS Statistical Package version 28 or later. Adverse effects and serious adverse effects will be tabulated and listed, by the treatment group.

### Treatment discontinuation

Participants who discontinue trial treatment will remain in the trial and trial procedures and scheduled visits will be conducted where possible.

### Post-trial care

At the end of the trial, participants will be unblinded and offered the option of either continuing their randomized treatment or changing to either standard antihypertensives or MRA treatment.

#### Ethics, trial registration and status

This trial protocol version 10, 19/12/23 has been approved by the local institution Human Research Ethics Committee, (RES-22-0000-044A) and has been registered with the Australian New Zealand Clinical Trials Registry (ACTRN12622000391774). Recruitment began in August 2022 and will continue until the sample size is reached.

Any further change to the protocol that affects the trial design or scientific intent will be submitted for approval to the Human Research Ethics Committee as an amendment.

## Discussion

The research findings of this study will improve our understanding of the pathophysiology and management of hypertension with low renin and may inform future hypertension guidelines.

De-identified results will be published in peer-reviewed journals and national and international conferences. Authorship will be offered to those who meet the National Health and Medical Research Council guidelines for authorship.

## Summary

### What is known about the topic

- Low-renin hypertension is common.
- Data from a meta-analysis showed that mineralocorticoid receptor antagonists were more effective in lowering blood pressure when compared to angiotensin-converting enzyme inhibitors and angiotensin receptor blockers. Key limitations of older studies include using high doses of mineralocorticoid receptor antagonists, inadequate exclusion of primary aldosteronism, short treatment duration and a lack of end-organ data.

### What this study adds

- This randomized controlled trial will address previous study limitations: primary aldosteronism will be excluded using gold-standard mass spectrometry measurement of aldosterone, participants will be treated for 48 weeks and data on markers of end-organ dysfunction will be collected.
- Recent evidence suggests that reversal of renin suppression is associated with better cardiovascular health in people with primary aldosteronism. This study will explore if a similar cardioprotective effect is seen in low-renin hypertension by titrating spironolactone to both blood pressure and renin concentration.

## References

[1] Australian Institute of Health and Welfare. Heart, stroke and vascular disease: Australian facts. Canberra: Australian Institute of Health and Welfare, 2024. https://www.aihw.gov.au/reports/heart-stroke-vascular-diseases/hsvd-facts.

[2] Olsen MH, Angell SY, Asma S, Boutouyrie P, Burger D, Chirinos JA, et al. A call to action and a lifecourse strategy to address the global burden of raised blood pressure on current and future generations: the Lancet Commission on hypertension. Lancet. 2016;388:2665–712. 27671667 10.1016/S0140-6736(16)31134-5

[3] Libianto R, Fuller PJ, Young MJ, Yang J. Primary aldosteronism is a public health issue: challenges and opportunities. J Hum Hypertens. 2020;34:478–86. 32341439 10.1038/s41371-020-0336-2

[4] Stowasser M, Gordon RD. Primary aldosteronism: changing definitions and new concepts of physiology and pathophysiology both inside and outside the kidney. Physiol Rev. 2016;96:1327–84. 27535640 10.1152/physrev.00026.2015

[5] Funder JW, Carey RM, Mantero F, Murad MH, Reincke M, Shibata H, et al. The management of primary aldosteronism: case detection, diagnosis, and treatment: an endocrine society clinical practice guideline. J Clin Endocrinol Metab. 2016;101:1889–916. 26934393 10.1210/jc.2015-4061

[6] Brown JM, Robinson-Cohen C, Luque-Fernandez MA, Allison MA, Baudrand R, Ix JH, et al. The spectrum of subclinical primary aldosteronism and incident hypertension: a cohort study. Ann Intern Med. 2017;167:630–41. 29052707 10.7326/M17-0882 PMC5920695

[7] Funder JW. Primary aldosteronism and low-renin hypertension: a continuum? Nephrol Dial Transplant. 2013;28:1625–7. 23535225 10.1093/ndt/gft052

[8] Baudrand R, Guarda FJ, Fardella C, Hundemer G, Brown J, Williams G, et al. Continuum of renin-independent aldosteronism in normotension. Hypertension. 2017;69:950–6. 28289182 10.1161/HYPERTENSIONAHA.116.08952 PMC5391287

[9] Shah SS, Zhang J, Gwini SM, Young MJ, Fuller PJ, Yang J. Efficacy and safety of mineralocorticoid receptor antagonists for the treatment of low-renin hypertension: a systematic review and meta-analysis. J Hum Hypertens. 2024;38:383–92. 10.1038/s41371-023-00891-1 PMC11076210 38200100

[10] Gabb GM, Mangoni AA, Anderson CS, Cowley D, Dowden JS, Golledge J, et al. Guideline for the diagnosis and management of hypertension in adults - 2016. Med J Aust. 2016;205:85–9. 27456450 10.5694/mja16.00526

[11] Whelton PK, Carey RM, Aronow WS, Casey DE Jr., Collins KJ, Dennison Himmelfarb C, et al. 2017 ACC/AHA/AAPA/ABC/ACPM/AGS/APhA/ASH/ASPC/NMA/PCNA guideline for the prevention, detection, evaluation, and management of high blood pressure in adults: a report of the American College of Cardiology/American Heart Association Task Force on Clinical Practice Guidelines. Circulation. 2018;138:e484–594. 30354654 10.1161/CIR.0000000000000596

[12] Ori Y, Chagnac A, Korzets A, Zingerman B, Herman-Edelstein M, Bergman M, et al. Regression of left ventricular hypertrophy in patients with primary aldosteronism/low-renin hypertension on low-dose spironolactone. Nephrol Dial Transplant. 2013;28:1787–93. 23378418 10.1093/ndt/gfs587

[13] Egan BM, Basile JN, Rehman SU, Davis PB, Grob CH 3rd, Riehle JF, et al. Plasma Renin test-guided drug treatment algorithm for correcting patients with treated but uncontrolled hypertension: a randomized controlled trial. Am J Hypertens. 2009;22:792–801. 19373213 10.1038/ajh.2009.63

[14] Australian Institute of Health and Welfare. Medicines for cardiovascular disease. Canberra: Australian Institute of Health and Welfare; 2017. https://www.aihw.gov.au/reports/heart-stroke-vascular-diseases/medicines-for-cardiovascular-disease.

[15] Chan AW, Tetzlaff JM, Gotzsche PC, Altman DG, Mann H, Berlin JA, et al. SPIRIT 2013 explanation and elaboration: guidance for protocols of clinical trials. BMJ. 2013;346:e7586. 23303884 10.1136/bmj.e7586 PMC3541470

[16] Weinberger MH, White WB, Ruilope LM, MacDonald TM, Davidson RC, Roniker B, et al. Effects of eplerenone versus losartan in patients with low-renin hypertension. Am Heart J. 2005;150:426–33. 16169319 10.1016/j.ahj.2004.12.005

[17] Hundemer GL, Leung AA, Kline GA, Brown JM, Turcu AF, Vaidya A. Biomarkers to guide medical therapy in primary aldosteronism. Endocr Rev. 2024;45:69–94. 37439256 10.1210/endrev/bnad024 PMC10765164

[18] Myers MG, Kaczorowski J, Dawes M, Godwin M. Automated office blood pressure measurement in primary care. Can Fam Physician. 2014;60:127–32. 24522674 PMC3922555

[19] Fuld S, Constantinescu G, Pamporaki C, Peitzsch M, Schulze M, Yang J, et al. Screening for primary aldosteronism by mass spectrometry versus immunoassay measurements of aldosterone: a prospective within-patient study. J Appl Lab Med. 2024;9:752–66. 10.1093/jalm/jfae017 38532521

[20] Stowasser M, Ahmed AH, Cowley D, Wolley M, Guo Z, McWhinney BC, et al. Comparison of seated with recumbent saline suppression testing for the diagnosis of primary aldosteronism. J Clin Endocrinol Metab. 2018;103:4113–24. 30239841 10.1210/jc.2018-01394

[21] National Health and Medical Research Council. Safety monitoring and reporting in clinical trials involving therapeutic goods. Australian Govererment; National Health and Medical Research Council; 2016. https://www.nhmrc.gov.au/about-us/publications/safety-monitoring-and-reporting-clinical-trials-involving-therapeutic-goods

[22] Norwegian Institute of Public Health, World Health Organization Collaborating Centre for Drug Statistics Methodology ATD/DDD Index; 2024. https://www.who.int/tools/atc-ddd-toolkit/methodology.

